# Dual-Time-Scale Cloud–Edge–End Collaborative Task Offloading for Multi-AGV Intelligent Warehousing in Industrial Internet of Things

**DOI:** 10.3390/s26123936

**Published:** 2026-06-21

**Authors:** Junjie Xue, Yuyi Huang, Yuheng Guo, Zhijian Lin, Bingxin Tian

**Affiliations:** 1 School of Advanced Manufacturing, Fuzhou University, Quanzhou 362251, China; 2 China Mobile Research Institute, Beijing 100053, China

**Keywords:** embodied intelligence, industrial Internet of Things, dual-time-scale optimization, cloud–edge–device collaborative computing, task offloading

## Abstract

In embodied-intelligence Industrial Internet of Things (IIoT), multi-AGV intelligent warehousing requires continuous processing of latency-sensitive tasks, such as environmental perception, inventory monitoring, and anomaly detection. Due to limited onboard computing capability and energy capacity, purely local execution can hardly satisfy real-time requirements, whereas fully cloud-based processing may incur excessive transmission delay and backhaul overhead. To address this issue, this paper investigates the joint optimization of AGV service-point migration and task offloading under a cloud-edge-end collaborative architecture. Considering the impact of service-point selection on wireless access, MEC resources, movement delay, and energy consumption, as well as the effect of offloading decisions on transmission, computation, and AGV-side energy cost, a dual-time-scale optimization model is formulated to minimize the long-term accumulated system delay while satisfying task latency and AGV energy constraints. To solve the resulting mixed discrete problem, a DPSO-MAPPO algorithm is proposed, where DPSO searches service-point plans satisfying movement and conflict constraints at the slow time scale, and MAPPO learns coordinated multi-AGV offloading policies at the fast time scale. The delay and energy feedback further enables coordination between the two types of decisions. Simulation results show that the proposed algorithm converges stably, reduces system delay by 13.55% compared with benchmark algorithms, and improves total energy consumption and energy-violation control.

## 1. Introduction

With the rapid development of the Industrial Internet of Things (IIoT) and embodied intelligence technologies, intelligent warehousing systems are evolving from conventional equipment automation toward the integration of perception, computation, decision making, and execution [1]. In this context, automated guided vehicles (AGVs) have become key mobile execution units that connect physical warehouse operations with information-driven decision-making systems [2,3]. Unlike conventional AGVs that mainly undertake material-handling tasks, AGVs in intelligent warehouses are generally equipped with cameras, radio-frequency identification (RFID) readers, and various onboard sensors. During inbound, outbound, inventory checking, inspection, and service-area operations, they can continuously collect information on cargo status, inventory state, traffic conditions, and equipment operating states, and further generate computation tasks such as cargo-status recognition, inventory-state monitoring, obstacle detection, anomaly identification, and operation control. These tasks are typically characterized by large data volumes, computation-intensive workloads, and stringent latency requirements. If the processing results cannot be returned in time, warehouse operation efficiency may degrade, task execution may be blocked, and operational safety may even be threatened. Therefore, an AGV-assisted intelligent warehousing system not only requires efficient mobility scheduling and task allocation mechanisms, but also needs a communication and computing architecture capable of supporting near-real-time data processing.

However, constrained by onboard computing capability, battery capacity, and vehicle-mounted resources, AGVs can hardly satisfy the real-time requirements of complex perception and control tasks through purely local computation. Offloading all tasks to remote cloud servers may also incur excessive transmission latency, backhaul burden, and service-latency fluctuations. By deploying computing resources close to the wireless access side, mobile edge computing (MEC) can alleviate the tension between limited terminal resources and long-distance cloud transmission, while cloud servers can still provide strong processing capability for computation-intensive or less latency-sensitive tasks [4]. Therefore, cloud-edge-end collaborative computing offers a feasible paradigm for AGV task processing in intelligent warehousing [5].

Nevertheless, in multi-AGV warehousing scenarios, task offloading decisions are tightly coupled with AGV service-point selection. On the one hand, the selected service point affects wireless channel quality, associated wireless access point (WAP)/MEC resources, movement delay, and mobility energy consumption, and may further cause service-point conflicts among multiple AGVs. On the other hand, the offloading mode in each time slot directly determines task upload latency, local/edge/cloud computation latency, and AGV-side energy consumption. Since AGV service-point migration usually occurs at a slower operational-stage time scale, whereas task generation and offloading decisions are made at a faster slot-level time scale, separate optimization may lead to myopic or energy-inefficient strategies. Therefore, for multi-AGV intelligent warehousing, it is necessary to jointly optimize slow-time-scale service-point migration and fast-time-scale cloud-edge-end task offloading, so as to reduce long-term accumulated system latency while satisfying task latency constraints and AGV energy budgets.

Existing studies have investigated AGV path planning, cloud-edge-end task offloading, MEC scheduling, and learning-based resource optimization from different perspectives. However, most of them focus on mobility scheduling, computation offloading, or resource allocation separately. The coupling between stage-wise AGV service-point migration and slot-level task offloading in multi-AGV intelligent warehousing has not been sufficiently investigated. A detailed discussion of related studies is provided in Section 2.

Motivated by the above limitations, this paper investigates the dual-time-scale joint optimization of AGV service-point migration and task offloading under a cloud-edge-end collaborative computing architecture for multi-AGV intelligent warehousing in embodied-intelligence IIoT. Unlike existing studies that mainly focus on fixed-route offloading or single-time-scale resource scheduling, this work jointly considers the coupling among stage-wise AGV service-point migration, wireless access-state variations, edge/cloud computing resources, local computing capability, and AGV energy constraints. The objective is to minimize the long-term accumulated system delay by jointly optimizing slow-time-scale service-point migration and fast-time-scale multi-type task offloading. The main contributions are summarized as follows:

1. A cloud-edge-end collaborative computing model is established for multi-AGV intelligent warehousing, and a dual-time-scale joint optimization problem of service-point migration and task offloading is formulated. The model captures stage-wise AGV migration among candidate service points, task generation during service periods, wireless transmission, local/edge/cloud computation, and AGV-side energy consumption, including movement, uplink transmission, and local computing energy. At the slow time scale, each AGV selects its next service point under candidate-point, maximum movement distance, and multi-AGV service-conflict constraints. At the fast time scale, each AGV makes offloading decisions among local, edge, and cloud execution according to task attributes, communication states, MEC resources, and residual energy. The problem minimizes the long-term accumulated system delay subject to task latency and AGV energy-budget constraints, thereby characterizing the coupling between mobility decisions and computation offloading in multi-AGV intelligent warehousing.
2. A DPSO-MAPPO dual-time-scale solution algorithm is proposed. For the discrete combinatorial structure of service-point selection at the slow time scale, discrete particle swarm optimization (DPSO) is employed to search feasible migration plans, with feasibility correction for movement-distance and service-conflict constraints. For fast-time-scale multi-AGV cooperative offloading, multi-agent proximal policy optimization (MAPPO) is adopted with a centralized-training and decentralized-execution mechanism to learn offloading policies under decentralized observations. By feeding the delay and energy information obtained from MAPPO into the DPSO fitness evaluation, the proposed algorithm realizes coordinated optimization between service-point planning and task offloading.
3. Extensive numerical simulations are conducted to verify the effectiveness of the proposed DPSO-MAPPO algorithm. The evaluation covers convergence behavior, system scale, time-scale parameter, MAPPO training parameters, DPSO population size, number of AGVs, number of candidate service points, and task data size, with comparisons against Random + MAPPO, DPSO + Dueling DQN, and Random + Greedy benchmarks. Results show that the proposed method converges stably and generates effective AGV service-point migration trajectories and cloud-edge-end offloading strategies. In a typical setting, it reduces system delay by 13.55% over the benchmarks and achieves better total energy consumption and energy-violation control.

The remainder of this paper is organized as follows. Section 2 reviews related studies on AGV scheduling, MEC scheduling, cloud-edge-end task offloading, and learning-based optimization methods. Section 3 presents the system model and problem formulation. Section 4 introduces the proposed DPSO–MAPPO dual-timescale joint solution algorithm. Section 5 provides numerical simulations and analyzes the convergence performance and optimization effectiveness of the proposed algorithm. Finally, Section 6 concludes this paper.

## 2. Related Works

### 2.1. AGV Scheduling and Service-Point Planning

Regarding AGV path planning and multi-AGV cooperative scheduling, recent studies mainly focus on real-time collaborative operation in large-scale warehouses, path-conflict avoidance, dynamic replanning, and task-completion-time reduction. Liu et al. investigated real-time collaborative operation of multiple AGVs in large-scale intelligent warehouses, aiming to improve multi-vehicle coordination efficiency in high-throughput warehousing environments [6]. Chen et al. studied AGV path planning and optimization in autonomous port management and proposed an ensemble reinforcement learning-based path-planning framework to enhance AGV decision-making capability in complex operational environments [7]. Wang et al. proposed a dynamic multi-AGV path-planning method based on an optimal time-reuse strategy, which realizes dynamic replanning and conflict-free movement by modeling point conflicts [8]. Li et al. developed a dynamic AGV scheduling model for special operational cases in matrix production workshops to improve the adaptability of AGV scheduling under production constraints [9]. These studies provide effective insights into multi-AGV path planning and cooperative scheduling. However, their optimization objectives mainly focus on movement-related aspects, such as travel paths, traffic conflicts, and operation-completion efficiency, while generally neglecting the perception and computation tasks generated by AGVs at different service points, as well as the impact of service-point selection on wireless access quality, edge computing resources, and subsequent task-offloading performance.

### 2.2. MEC Scheduling and Cloud-Edge-End Task Offloading

Regarding computation offloading in the Industrial Internet of Things, edge computing and cloud–edge–end collaborative computing have been widely adopted to alleviate the latency caused by insufficient computing capability of terminal devices and long-distance transmission to remote clouds. Laili et al. proposed a DSAC-configured differential evolution-based task scheduling method for cloud–edge–device collaborative manufacturing environments to improve task scheduling efficiency across multi-layer computing resources [10]. Wu et al. investigated the joint optimization of multi-AGV task offloading and resource scheduling for mobile inspection services in smart factories, where MEC and D2D offloading are jointly exploited to reduce AGV energy consumption under latency, power, computing-capacity, and bandwidth constraints [11]. Liu et al. proposed the MATO algorithm for industrial IoT scenarios with multiple fixed-route AGVs, where weighted polling and DQN are adopted to optimize the offloading matching between AGVs and edge servers, thereby alleviating resource competition caused by multiple AGVs accessing the same edge server [12]. Shi et al. further considered distributed AGV systems with multiple MEC servers and proposed a Transformer-based MADRL method, which models inter-AGV relationships to mitigate transmission conflicts and channel-access conflicts caused by concurrent AGV offloading [13]. These studies demonstrate that cloud–edge–end collaborative offloading can effectively improve task-processing efficiency in IIoT and AGV scenarios. However, most existing works still assume that AGV routes, access sequences, or network topologies are known to some extent, and mainly focus on offloading decisions, resource scheduling, or channel access under given mobility patterns, without jointly optimizing AGV service-point migration and task offloading in a dual-timescale manner.

Recent studies on mobile edge computing (MEC) scheduling have further investigated how to coordinate task offloading, resource allocation, and queue stability under dynamic network conditions. Bi et al. proposed a Lyapunov-guided deep reinforcement learning framework for online computation offloading in MEC networks, where stochastic task arrivals, time-varying wireless channels, long-term queue stability, and average power constraints are jointly considered [14]. Hao et al. developed EdgeTimer, an adaptive multi-timescale scheduling framework for MEC systems, which uses hierarchical deep reinforcement learning to dynamically adjust scheduling timescales across different edge-cloud layers and improve the tradeoff between service delay and operation cost [15]. More recently, Fan et al. studied energy-constrained multimodal ISAC systems and proposed a Lyapunov-driven heterogeneous mixture-of-experts architecture to balance tracking accuracy, computing congestion, and long-term energy budgets [16]. These works demonstrate the importance of dynamic scheduling and resource coordination in MEC systems. However, most of them mainly focus on communication-computation scheduling, queue stability, or multi-layer resource management, while the coupling between AGV service-point migration and slot-level cloud-edge-end task offloading in intelligent warehousing remains insufficiently explored.

### 2.3. Learning-Based Optimization Methods

From the perspective of solution methods, existing task offloading and resource scheduling problems are commonly solved using heuristic optimization, deep reinforcement learning, and multi-agent reinforcement learning [17,18]. Xue et al. applied an improved particle-swarm genetic algorithm to the multi-AGV multi-task assignment scheduling problem, where heuristic search is used to obtain high-quality task allocation schemes [19]. Cai et al. proposed a multitask multiobjective deep reinforcement learning method for IIoT task offloading to optimize computation offloading performance under dynamic task and resource states [20]. Gao et al. introduced multi-agent reinforcement learning for large-scale computation offloading in heterogeneous multi-access edge computing, improving the adaptability of distributed offloading decisions in large-scale scenarios [21]. Zhang et al. further investigated cooperative partial task offloading and resource allocation in IIoT, and proposed a decentralized multi-agent deep reinforcement learning method to support cooperative decision making among multiple devices [22]. These methods provide effective solutions for dynamic task offloading and multi-agent cooperative optimization. However, they mainly focus on task offloading, resource allocation, or AGV scheduling at a single timescale. Directly incorporating both AGV service-point selection and slot-level task offloading into the reinforcement-learning action space would significantly enlarge the state–action space and make training convergence difficult. Therefore, a dual-timescale solution method that combines discrete search with multi-agent learning is needed to separately handle slow-timescale service-point migration decisions and fast-timescale task offloading decisions, while coordinating them through latency and energy feedback.

## 3. System Model and Problem Formulation

### 3.1. System Architecture

**Figure 1:**
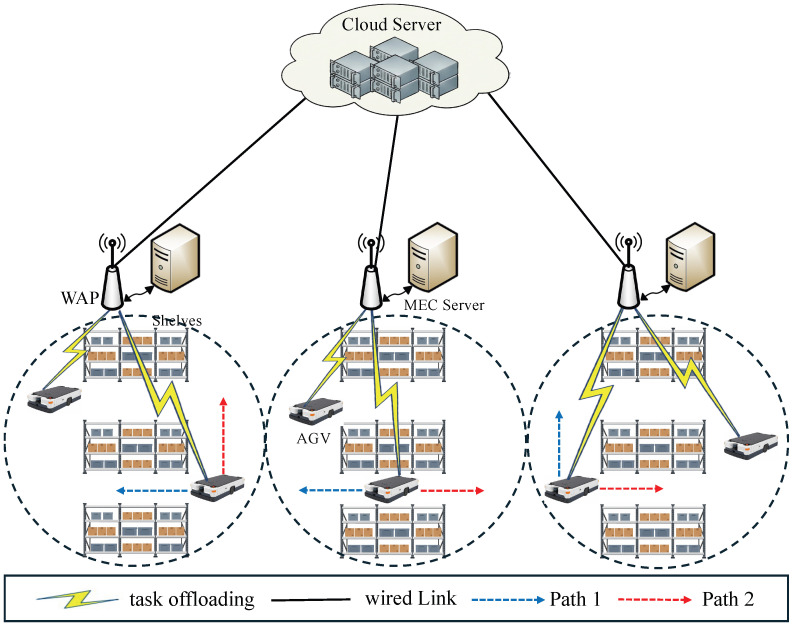
Illustration of the cloud-edge-end collaborative multi-AGV intelligent warehousing scenario.

This paper considers an embodied-intelligence industrial Internet of Things (IIoT) intelligent warehousing scenario and constructs a cloud–edge–end collaborative computing framework, as shown in Figure 1. The system consists of multiple AGVs that execute environmental perception and operation-control tasks, multiple MEC servers providing edge computing resources, and a cloud server [23,24]. Each AGV is equipped with cameras and other onboard sensors to continuously acquire environmental information and operational states during warehouse operations, and generates corresponding computation tasks, such as cargo-status perception, inventory-status monitoring, and anomaly detection in service areas [25]. Since such tasks are typically featured by large data volumes, computation-intensive workloads, and stringent delay requirements, relying solely on cloud processing may incur substantial data-return overhead and unstable latency. Therefore, MEC servers are deployed at the access side to provide near-end computing support. Let the set of AGVs be I={1,2,…,i,…,|I|}, the set of MEC servers be M={1,2,…,m,…,|M|}, and denote the cloud server by C. AGVs access the network via wireless access points (WAPs), and task data can be offloaded through WAPs to either an edge-side MEC server or the cloud server for processing.

To capture the different decision frequencies between the stage-wise service-point migration of AGVs and the slot-level task processing, we adopt a two-time-scale modeling approach. Specifically, the system operation is divided into discrete small time-scale slots, with the slot set denoted by T={1,2,…,t,…,|T|} and each slot having duration Δ. Meanwhile, every *H* consecutive small time-scale slots form one large time-scale decision interval, i.e., the *n*-th large time-scale decision is triggered when t=nH, where n=1,2,…. At the large time scale, each AGV determines its next service point; between two adjacent large time-scale decision instants, the AGV stays within the current service area to process tasks, and in each small time-scale slot it generates tasks and makes the corresponding offloading decisions. Let the set of task types be K={1,2,…,k,…,|K|}, where *k* is the task-type index. The task of type *k* generated by AGV *i* in slot *t* is denoted by $\tau_{i,k}\left(t\right)$. For each task type *k*, $\tau_{i,k}\left(t\right)$ represents a slot-level aggregated computation task generated by AGV *i* in slot *t*, with time-varying workload attributes. The task attributes are denoted by $\Omega_{i,k}\left(t\right)=\left(D_{i,k}\left(t\right),C_{i,k}\left(t\right),T_{k}^{\max}\right)$, where $D_{i,k}\left(t\right)$ is the input data size, $C_{i,k}\left(t\right)$ is the required CPU cycles per bit, and $T_{k}^{\max}$ is the maximum tolerable delay for tasks of type *k*.

### 3.2. Mobility Model

We consider AGVs performing embodied-intelligence operations in a warehouse environment. Each AGV migrates between candidate service points in a stage-wise manner, and after arriving at a target service point, it stays for *H* consecutive small time-scale slots to complete task generation and processing within the corresponding service stage [26]. Let the service-point position of AGV *i* at the *n*-th large time-scale decision interval be $p_{i}\left(n\right)=\left(x_{i}\left(n\right),y_{i}\left(n\right)\right)$, and let $S_{i}$ denote its set of candidate service points, such that $p_{i}\left(n\right)\in S_{i}$. Considering that multiple AGVs coexist in the system, selecting the same service point may lead to operation conflicts. Therefore, we impose that the service-point sets of different AGVs do not overlap, i.e., $P_{i}\cap P_{i^{\prime }}=⌀,\;\forall i\neq i^{\prime }$, where $P_{i}=\left\{p_{i}\left(n\right)\right\}$ denotes the service-point set of AGV *i* over the operation horizon.

Accordingly, when AGV *i* moves from the current service point $p_{i}\left(n\right)$ to the next service point $p_{i}\left(n+1\right)$ with speed $v_{i}$, the moving latency is defined as

(1) $$T_{i}^{\mathrm{mo}}\left(n\right)=\frac{\sqrt{{\left(x_{i}\left(n+1\right)-x_{i}\left(n\right)\right)}^{2}+{\left(y_{i}\left(n+1\right)-y_{i}\left(n\right)\right)}^{2}}}{v_{i}}.$$

Furthermore, we adopt a constant-traction power model to characterize the mobility energy consumption of AGV *i*, and the corresponding moving energy consumption is expressed as

(2) $$E_{i}^{\mathrm{mv}}\left(n\right)=P_{i}^{\mathrm{mv}}T_{i}^{\mathrm{mo}}\left(n\right),$$

where $P_{i}^{\mathrm{mv}}$ is the constant moving power of AGV *i*. We assume a sequential service process in which task generation, uploading, and computation are performed only after the AGV arrives at the selected service point; therefore, no task data upload is carried out during migration.

### 3.3. Communication Model

As discussed above, each AGV performs task uploading and processing only at service points; therefore, the communication process at the small time scale takes place within the current service stage. For task $\tau_{i,k}\left(t\right)$, if it is executed locally, at the edge, or at the cloud, the corresponding offloading decision variables are denoted by $x_{i,k}^{\mathrm{loc}}\left(t\right)$, $x_{i,k}^{\mathrm{mec}}\left(t\right)$, and $x_{i,k}^{\mathrm{cld}}\left(t\right)$, respectively.

In a two-dimensional Cartesian coordinate system, we assume that in each time slot the AGV associates with the nearest wireless access point (WAP) [27]. Let the coordinates of the WAP associated with AGV *i* in time slot *t* be $\left(x_{i}^{a}\left(t\right),y_{i}^{a}\left(t\right)\right)$. Considering distance-dependent propagation and path loss, the wireless channel gain of AGV *i* is given by

(3) $$h_{i}\left(t\right)=\frac{X}{{\left(x_{i}\left(t\right)-x_{i}^{a}\left(t\right)\right)}^{2}+{\left(y_{i}\left(t\right)-y_{i}^{a}\left(t\right)\right)}^{2}},$$

where *X* is the channel-gain constant at a reference distance of 1 m. Accordingly, the uplink transmission rate of AGV *i* in time slot *t* can be written as

(4) $$R_{i}\left(t\right)=B\log_{2}\;\left(1+\frac{P_{i}^{\mathrm{tx}}h_{i}\left(t\right)}{n_{0}}\right),$$

where *B* is the uplink bandwidth allocated to the AGV, $P_{i}^{\mathrm{tx}}$ is the transmit power of AGV *i*, and $n_{0}$ is the receiver noise power.

When task $\tau_{i,k}\left(t\right)$ is offloaded for edge execution, the wireless uplink transmission latency is

(5) $$T_{i,k}^{\mathrm{up},E}\left(t\right)=\frac{x_{i,k}^{\mathrm{mec}}\left(t\right)D_{i,k}\left(t\right)}{R_{i}\left(t\right)}.$$

When task $\tau_{i,k}\left(t\right)$ is offloaded for cloud execution, the task data is first uploaded to the associated WAP via the wireless link and then forwarded to the cloud server through the backhaul link, and thus the uplink transmission latency is expressed as

(6) $$T_{i,k}^{\mathrm{up},C}\left(t\right)=x_{i,k}^{\mathrm{cld}}\left(t\right)\left(\frac{D_{i,k}\left(t\right)}{R_{i}\left(t\right)}+\frac{D_{i,k}\left(t\right)}{R_{i}^{\mathrm{bh}}\left(t\right)}\right),$$

where $R_{i}^{\mathrm{bh}}\left(t\right)$ denotes the equivalent backhaul transmission rate from the WAP associated with AGV *i* to the cloud server.

Accordingly, the wireless uplink energy consumption at the AGV side can be written as

(7) $$E_{i,k}^{\mathrm{up}}\left(t\right)=P_{i}^{\mathrm{tx}}\cdot \frac{\left(x_{i,k}^{\mathrm{mec}}\left(t\right)+x_{i,k}^{\mathrm{cld}}\left(t\right)\right)D_{i,k}\left(t\right)}{R_{i}\left(t\right)}.$$

Since the size of the returned result is typically much smaller than that of the input data, we neglect the transmission latency and reception energy consumption in the result-return stage.

### 3.4. Computation Model

Due to the limited computing capability at the AGV side, task $\tau_{i,k}\left(t\right)$ can be executed locally, at an edge-side MEC server, or at the cloud. When the task is executed locally on AGV *i*, the corresponding computation latency is

(8) $$T_{i,k}^{\mathrm{cmp},\mathrm{loc}}\left(t\right)=x_{i,k}^{\mathrm{loc}}\left(t\right)\frac{C_{i,k}\left(t\right)D_{i,k}\left(t\right)}{f_{i}},$$

where $f_{i}$ denotes the computing frequency of AGV *i*.

When the task is offloaded for edge execution, we assume that it is completed at the MEC server currently associated with the AGV, and the corresponding edge computation latency is

(9) $$T_{i,k}^{\mathrm{cmp},E}\left(t\right)=x_{i,k}^{\mathrm{mec}}\left(t\right)\frac{C_{i,k}\left(t\right)D_{i,k}\left(t\right)}{f_{m_{i}\left(t\right)}},$$

where $f_{m_{i}\left(t\right)}$ denotes the computing frequency of the MEC server associated with AGV *i* in time slot *t*.

When the task is offloaded for cloud execution, the cloud computation latency is given by

(10) $$T_{i,k}^{\mathrm{cmp},C}\left(t\right)=x_{i,k}^{\mathrm{cld}}\left(t\right)\frac{C_{i,k}\left(t\right)D_{i,k}\left(t\right)}{f_{C}}.$$

Here, $f_{C}$ denotes the computing frequency of the cloud server.

For the energy model, we consider only the local computation energy consumption at the AGV side. The computation energy consumption of task $\tau_{i,k}\left(t\right)$ when executed locally can be written as

(11) $$E_{i,k}^{\mathrm{cmp},\mathrm{loc}}\left(t\right)=\kappa_{i}f_{i}^{3}T_{i,k}^{\mathrm{cmp},\mathrm{loc}}\left(t\right),$$

where $\kappa_{i}$ is the energy coefficient of AGV *i*. Since the computation energy consumption at the edge and cloud is supplied by the infrastructure, it is not included in the AGV energy budget in this paper.

### 3.5. Latency and Energy Consumption Model

The uplink transmission latency of task $\tau_{i,k}\left(t\right)$ consists of the edge-uplink latency and the cloud-uplink latency, i.e.,

(12) $$T_{i,k}^{\mathrm{up}}\left(t\right)=T_{i,k}^{\mathrm{up},E}\left(t\right)+T_{i,k}^{\mathrm{up},C}\left(t\right).$$

Correspondingly, the computation latency of task $\tau_{i,k}\left(t\right)$ consists of local computation, edge computation, and cloud computation, i.e.,

(13) $$T_{i,k}^{\mathrm{cmp}}\left(t\right)=T_{i,k}^{\mathrm{cmp},\mathrm{loc}}\left(t\right)+T_{i,k}^{\mathrm{cmp},E}\left(t\right)+T_{i,k}^{\mathrm{cmp},C}\left(t\right).$$

Since task uploading and computation are performed after the AGV arrives at the current service point, the end-to-end processing latency of task $\tau_{i,k}\left(t\right)$ at the small time scale can be expressed as

(14) $$T_{i,k}^{e2e}\left(t\right)=T_{i,k}^{\mathrm{up}}\left(t\right)+T_{i,k}^{\mathrm{cmp}}\left(t\right).$$

Furthermore, considering the combined impact of the small time-scale task-processing latency and the large time-scale service-point migration latency over the entire operation horizon, the long-term accumulated system latency is defined as

(15) $$T^{\mathrm{sys}}=\sum_{n=1}^{N}\sum_{i\in I}T_{i}^{\mathrm{mo}}\left(n\right)+\sum_{t\in T}\sum_{i\in I}\sum_{k\in K}\omega_{k}\;T_{i,k}^{e2e}\left(t\right),$$

where *N* denotes the number of large time-scale decision intervals in the operation horizon, and $\omega_{k}$ denotes the latency weight of task type *k*.

For energy consumption, we consider both the mobility energy at the large time scale and the task uplink energy and local computation energy at the small time scale [28]. Accordingly, the total energy consumption of AGV *i* over the operation horizon can be written as

(16) $$E_{i}^{\mathrm{tot}}=\sum_{n=1}^{N}E_{i}^{\mathrm{mv}}\left(n\right)+\sum_{t\in T}\sum_{k\in K}\left(E_{i,k}^{\mathrm{up}}\left(t\right)+E_{i,k}^{\mathrm{cmp},\mathrm{loc}}\left(t\right)\right).$$

### 3.6. Dual-Time-Scale Joint Optimization Problem

This subsection formulates the dual-time-scale joint decision problem in the intelligent warehousing scenario. The objective is to minimize the long-term accumulated system latency while satisfying the energy budget constraint of each AGV. At the small time scale, we optimize the task-offloading decision variables $X=\{x_{i,k}^{\mathrm{loc}}\left(t\right),x_{i,k}^{\mathrm{mec}}\left(t\right),x_{i,k}^{\mathrm{cld}}\left(t\right)\}$; at the large time scale, we optimize the service-point migration decision variables $P=\{p_{i}\left(n\right)\}$. Accordingly, the dual-time-scale joint optimization problem of interest can be expressed as

(17a) $$P1:\;\min_{X,P}\;T^{\mathrm{sys}}$$

(17b) $$s.t.\;x_{i,k}^{\mathrm{loc}}\left(t\right)+x_{i,k}^{\mathrm{mec}}\left(t\right)+x_{i,k}^{\mathrm{cld}}\left(t\right)=1,\;\forall i,k,t,$$

(17c) $$T_{i,k}^{e2e}\left(t\right)\leq T_{k}^{\max},\;\forall i,k,t,$$

(17d) $$p_{i}\left(n\right)\in S_{i},\;\forall i,n,$$

(17e) $$0<∥p_{i}\left(n+1\right)-p_{i}\left(n\right)∥\leq d_{i}^{\max},\;\forall i,n,$$

(17f) $$P_{i}\cap P_{i^{\prime }}=⌀,\;\forall i\neq i^{\prime },$$

(17g) $$E_{i}^{\mathrm{tot}}\leq E_{i}^{\max},\;\forall i,$$

(17h) $$x_{i,k}^{\mathrm{loc}}\left(t\right),\;x_{i,k}^{\mathrm{mec}}\left(t\right),\;x_{i,k}^{\mathrm{cld}}\left(t\right)\in \left\{0,1\right\},\;\forall i,k,t.$$

Here, constraint (17b) ensures that each task chooses one among local, edge, and cloud execution; constraint (17c) is the task latency constraint; constraints (17d)–(17f) characterize the feasibility of service points, the feasibility of stage-wise migration, and the operation-conflict avoidance among multiple AGVs, respectively; constraint (17g) is the energy budget constraint of each AGV; and constraint (17h) specifies the domains of the offloading decision variables.

## 4. DPSO–MAPPO Dual-Time-Scale Joint Solution Algorithm

Problem P1 involves both the large time-scale service-point migration decisions of AGVs and the small time-scale task offloading decisions [19,21]. The former affects the wireless access conditions, moving latency, and mobility energy consumption during the subsequent service period, while the latter determines the execution mode of each task, i.e., local, edge, or cloud execution. Since these two types of decisions differ in time scale and variable type and are coupled through the system latency objective and the energy constraints, directly solving P1 is challenging.

**Figure 2:**
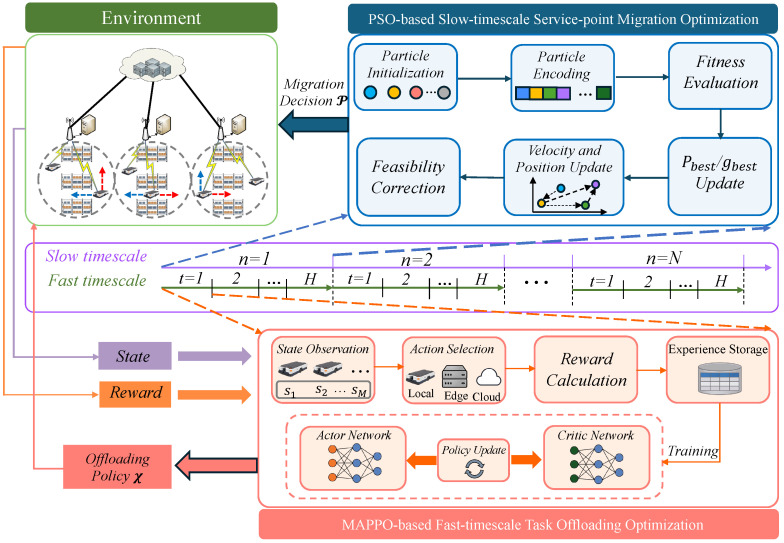
Framework of the proposed PSO–MAPPO dual-timescale service-point migration and task offloading method.

To address this issue, we develop a DPSO–MAPPO dual-time-scale joint solution framework, as illustrated in Figure 2. At the large time scale, the service-point selection is a discrete decision problem over a finite set of candidate service points, which is solved by a discrete particle swarm optimization (DPSO) algorithm. At the small time scale, the task offloading of multiple AGVs is modeled as a multi-agent decision-making problem, and MAPPO is adopted to learn the offloading policy. Specifically, the outer-layer DPSO generates service-point migration plans, while the inner-layer MAPPO performs task offloading under a given loitering plan. The accumulated latency and the satisfaction of energy constraints are then fed back to the outer layer as evaluation criteria.

### 4.1. Large-Time-Scale Service-Point Migration Optimization via DPSO

At the large time scale, each AGV selects its next service point from a candidate service-point set. Since $p_{i}\left(n+1\right)\in S_{i}$ is a discrete selection variable, we employ an integer-encoded discrete particle swarm optimization (DPSO) algorithm to solve the service-point migration subproblem [19]. Let the DPSO population size be *D*. The position of the *d*-th particle in the *r*-th search iteration is denoted by $z_{d}^{r}=\left[z_{d,1}^{r},z_{d,2}^{r},\ldots ,z_{d,|I|}^{r}\right]$, where $z_{d,i}^{r}\in \left\{1,2,\ldots ,\right|S_{i}\left|\right\}$ indicates that AGV *i* selects the $z_{d,i}^{r}$-th service point in $S_{i}$. Accordingly, the next-loitering plan corresponding to particle *d* can be expressed as

(18) $$p_{i}\left(n+1\right)=S_{i}\;\left[z_{d,i}^{r}\right],\;\forall i\in I.$$

To evaluate the quality of different service-point plans, we adopt the system accumulated latency under a given plan as the primary fitness metric, and impose penalties for violations of the energy constraints. The fitness function of particle *d* is defined as

(19) $$F_{d}^{r}=T_{\mathrm{DPSO}}^{\mathrm{sys}}+\lambda_{E}\sum_{i\in I}\max\;\left(E_{i}^{\mathrm{tot}}-E_{i}^{\max},0\right),$$

where $T_{\mathrm{DPSO}}^{\mathrm{sys}}$ denotes the system accumulated latency obtained by the small-time-scale offloading policy under the loitering plan represented by the current particle, and $\lambda_{E}$ is the penalty coefficient for energy-budget violations. A smaller fitness value indicates a better service-point plan in terms of latency and energy feasibility.

In the *r*-th search iteration, let the velocity of particle *d* be $v_{d}^{r}=\left[v_{d,1}^{r},v_{d,2}^{r},\ldots ,v_{d,|I|}^{r}\right]$, and let its personal best position and the global best position be $p_{d,\mathrm{best}}^{r}$ and $g_{\mathrm{best}}^{r}$, respectively. The particle velocity is updated as

(20) $$v_{d}^{r+1}=\omega v_{d}^{r}+c_{1}\rho_{1}\left(p_{d,\mathrm{best}}^{r}-z_{d}^{r}\right)+c_{2}\rho_{2}\left(g_{\mathrm{best}}^{r}-z_{d}^{r}\right),$$

where ω is the inertia weight, $c_{1}$ and $c_{2}$ are learning factors, and $\rho_{1}$ and $\rho_{2}$ are random numbers in [0,1]. Since the service-point selection is discrete, the particle position is updated with an integer projection as

(21) $$z_{d,i}^{r+1}=\Pi_{S_{i}}\;\left(\mathrm{round}\;\left(z_{d,i}^{r}+v_{d,i}^{r+1}\right)\right),$$

where round(·) denotes the rounding operation, and $\Pi_{S_{i}}\left(\cdot \right)$ projects the updated index onto the admissible index range of candidate service points in $S_{i}$.

To ensure that the service-point plan represented by each particle satisfies the mobility constraints and service-point conflict constraints, we perform feasibility corrections after each position update. Specifically, if $p_{i}\left(n+1\right)\notin S_{i}$, it is remapped to a feasible candidate point in $S_{i}$; if $∥p_{i}\left(n+1\right)-p_{i}\left(n\right)∥>d_{i}^{\max}$ or $∥p_{i}\left(n+1\right)-p_{i}\left(n\right)∥=0$, the next service point is reselected from candidates satisfying the migration-distance constraint; if multiple AGVs select the same service point, the selection with a better fitness contribution is retained, while the remaining conflicting AGVs are reassigned to feasible service points. After the above updates and corrections, DPSO yields a large-time-scale service-point migration plan, which provides the location and access-state inputs for the subsequent small-time-scale task-offloading optimization.

### 4.2. Small-Time-Scale Task Offloading Optimization via MAPPO

Given a large-time-scale loitering plan P, each AGV makes offloading decisions during the service period according to the current tasks, communication conditions, available edge computing resources, and its remaining energy budget. We model each AGV as an agent and formulate the small-time-scale task offloading process as a multi-agent MDP, denoted by (S,A,r,γ), where S, A, *r*, and γ represent the state space, action space, reward function, and discount factor, respectively [20,21].

At time slot *t*, the local state of AGV *i* is defined as

(22) $$s_{i}\left(t\right)={\left\{\Omega_{i,k}\left(t\right),\;p_{i}\left(n\right),\;R_{i}\left(t\right),\;f_{m_{i}\left(t\right)},\;{\bar{E}}_{i}\left(t\right)\right\}}_{k\in K},$$

where ${\bar{E}}_{i}\left(t\right)$ denotes the remaining energy budget of AGV *i* at time slot *t*. The global state is the aggregation of all local states, i.e.,

(23) $$s\left(t\right)={\left\{s_{i}\left(t\right)\right\}}_{i\in I}.$$

The action of AGV *i* at time slot *t* represents its offloading choice for each task type, i.e.,

(24) $$a_{i}\left(t\right)={\left\{x_{i,k}^{\mathrm{loc}}\left(t\right),\;x_{i,k}^{\mathrm{mec}}\left(t\right),\;x_{i,k}^{\mathrm{cld}}\left(t\right)\right\}}_{k\in K}.$$

For any task $\tau_{i,k}\left(t\right)$, the action satisfies $x_{i,k}^{\mathrm{loc}}\left(t\right)+x_{i,k}^{\mathrm{mec}}\left(t\right)+x_{i,k}^{\mathrm{cld}}\left(t\right)=1$. The joint action is given by

(25) $$a\left(t\right)={\left\{a_{i}\left(t\right)\right\}}_{i\in I}.$$

To guide the agents to reduce task processing latency while satisfying the constraints, the instantaneous reward at the small time scale is defined as

(26) $$\begin{array}{cc} r(t) & =-\sum_{i\in I}\sum_{k\in K}\omega_{k}T_{i,k}^{e2e}\left(t\right)\\ \; & \;-\lambda_{T}\sum_{i\in I}\sum_{k\in K}\max\;\left(T_{i,k}^{e2e}\left(t\right)-T_{k}^{\max},0\right)\\ \; & \;-\lambda_{E}\sum_{i\in I}\max\;\left(E_{i}^{\mathrm{used}}\left(t\right)-E_{i}^{\max},0\right), \end{array}$$

where $\lambda_{T}$ and $\lambda_{E}$ are penalty coefficients, and $E_{i}^{\mathrm{used}}\left(t\right)$ denotes the accumulated energy consumption of AGV *i* up to time slot *t*. The state transition is jointly determined by task generation, channel variations, offloading actions, and energy consumption.

To solve the above multi-agent MDP, we adopt MAPPO with centralized training and decentralized execution [29]. During training, the critic network evaluates the state value based on the global state s(t), while each actor network outputs an offloading action based on the corresponding local state $s_{i}\left(t\right)$. During execution, each AGV makes its offloading decision independently according to its own local observation.

Let the actor and critic network parameters be θ and ϕ, respectively. Under the old policy $\pi_{\theta_{\mathrm{old}}}$ and the current policy $\pi_{\theta }$, the policy probability ratio for AGV *i* at time slot *t* is defined as

(27) $$\beta_{i}\left(t;\theta \right)=\frac{\pi_{\theta }\;\left(a_{i}\left(t\right)|s_{i}\left(t\right)\right)}{\pi_{\theta_{\mathrm{old}}}\;\left(a_{i}\left(t\right)|s_{i}\left(t\right)\right)}.$$

Based on sampled trajectories, the discounted return at time slot *t* is defined as

(28) $$G\left(t\right)=\sum_{\ell =t}^{T}\gamma^{\ell -t}r\left(\ell \right).$$

Accordingly, the advantage estimate is

(29) $$\hat{A}\left(t\right)=G\left(t\right)-V_{\phi }\;\left(s(t)\right).$$

The MAPPO actor networks are updated using the PPO clipped objective [30]. For AGV *i*, the actor optimization objective is

(30) $$J_{i}\left(\theta \right)=E\left[\min\left(\beta_{i}\left(t;\theta \right)\hat{A}\left(t\right),\;\mathrm{clip}\;\left(\beta_{i}\left(t;\theta \right),1-\epsilon ,1+\epsilon \right)\hat{A}\left(t\right)\right)\right],$$

where ϵ is the clipping coefficient. All AGV actor networks update their policies according to (30). The critic network is updated by minimizing the value estimation error, with the loss function given by

(31) $$L\left(\phi \right)=E\;\left[{\left(G\left(t\right)-V_{\phi }\;\left(s(t)\right)\right)}^{2}\right].$$

In each training iteration, all AGVs interact with the environment under the current policies and collect {s(t),a(t),r(t),s(t+1)} into an experience buffer; then, the actor and critic networks are updated using mini-batch samples. After training, MAPPO yields the small-time-scale task offloading policy, which provides task-processing decisions under a given loitering plan.

### 4.3. DPSO–MAPPO Joint Solving Procedure

To jointly solve the large-time-scale service-point migration decisions and the small-time-scale task offloading decisions, we develop a DPSO–MAPPO joint solving framework. DPSO searches for candidate service-point migration plans at the large time scale; given a loitering plan, MAPPO performs multi-AGV task offloading decisions at the small time scale, and feeds back the resulting system cumulative latency and energy-budget satisfaction to DPSO for particle fitness evaluation.

Specifically, within the *n*-th large-time-scale decision interval, DPSO first initializes particle positions and velocities, and maps each particle to a candidate loitering plan. To ensure the comparability of particle fitness values, each particle is evaluated via simulation starting from the same system state at the current large-time-scale decision instant. For a given particle, the system determines the current AGV locations, wireless access conditions, and edge computing conditions according to the corresponding loitering plan. Then, MAPPO executes small-time-scale offloading decisions during the service period using the current policy, collects per-slot offloading actions, rewards, and state-transition samples, and computes the system cumulative latency and energy consumption associated with this particle. DPSO then calculates the particle fitness according to (19), updates the personal-best and global-best positions, and generates new candidate loitering plans via position updating and feasibility repair.

It should be noted that MAPPO is not retrained or updated during each particle evaluation. During the fitness evaluation of all DPSO particles within the same large-time-scale decision interval, the MAPPO policy parameters are kept fixed, so that different candidate service-point plans are evaluated under the same offloading policy. Instead, MAPPO interacts at the small time scale using the fixed current policy and stores the generated samples into an experience buffer. After completing the DPSO search under the current large-time-scale decision interval, the actor and critic networks are updated using the collected samples.The above procedure is repeated across large-time-scale decision intervals until the training termination condition is satisfied. Finally, DPSO outputs the service-point migration plan, and MAPPO outputs the corresponding task offloading policy. Algorithm 1 summarizes the overall DPSO–MAPPO joint solving procedure.

### 4.4. Computational Complexity and Deployment Discussion

Let N=|I| denote the number of AGVs, K=|K| denote the number of task types, *D* denote the DPSO population size, $R_{\max}$ denote the maximum number of DPSO iterations, and *H* denote the number of small time-scale slots within each large time-scale decision interval. In the proposed DPSO–MAPPO framework, the main computational cost comes from the outer-layer DPSO particle evaluation and the inner-layer MAPPO offloading decision. For each large time-scale interval, DPSO evaluates *D* particles over $R_{\max}$ iterations, and each particle evaluation involves MAPPO-based offloading decisions for *N* AGVs over *H* small time-scale slots. Therefore, the dominant online evaluation complexity can be expressed as

$$O\left(R_{\max}DHNC_{\pi }\right),$$

where $C_{\pi }$ denotes the computational cost of one actor-network forward inference. Since the MAPPO actor network is a lightweight multilayer perceptron, the online inference cost is much lower than the offline training cost.

To evaluate the online execution efficiency, we measured the CPU-only inference latency of the trained MAPPO policy on the simulation platform. The actor network contains two hidden layers with 64 neurons each, and each AGV actor has 5705 trainable parameters. The measurement was conducted with 100 warm-up runs and 5000 repeated runs. The results are summarized in Table 1.
**Algorithm 1:** DPSO–MAPPO Dual-Timescale Joint Optimization Algorithm.
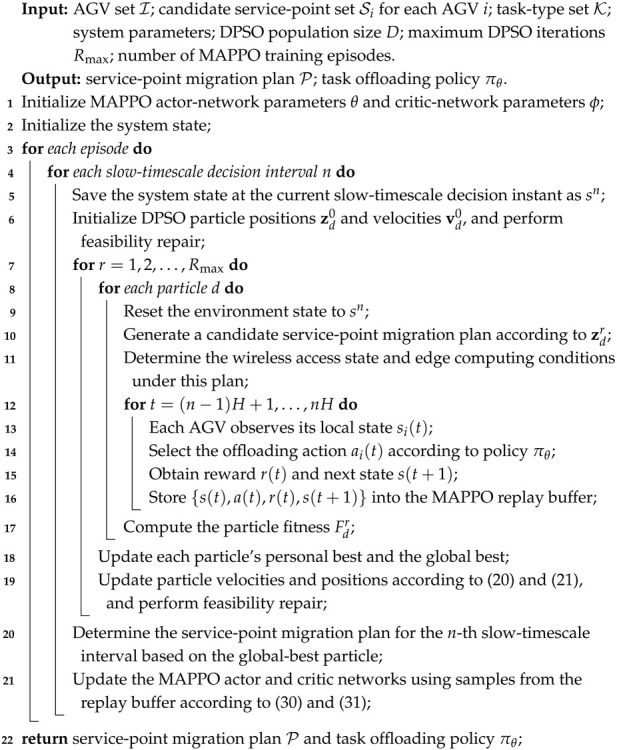


As shown in Table 1, the average latency for generating the joint offloading action of all AGVs is 0.2392 ms, and the average end-to-end online decision latency is 1.3793 ms. These values are much smaller than the duration of a small time-scale slot in the considered warehouse scheduling scenario. Therefore, the trained MAPPO policy can support real-time offloading decisions. In practical deployment, MAPPO training can be completed offline, while online execution only requires lightweight actor inference. The DPSO-based service-point optimization is performed at the larger time scale and can be executed by an edge scheduler or warehouse control server. Thus, the proposed DPSO–MAPPO framework is suitable for hierarchical deployment in intelligent warehousing systems.

## 5. Numerical Simulations

This section conducts numerical experiments to verify the effectiveness of the proposed DPSO–MAPPO dual-time-scale joint optimization algorithm and compares it with several baseline methods.

### 5.1. Simulation Parameter Settings

We consider an intelligent warehousing scenario consisting of |I|=4 AGVs, |M|=4 MEC servers, and one cloud server. The warehouse area contains 20 candidate service points, and the candidate service points of each AGV are given by its set $S_{i}$. The task-type set is denoted by K and includes three types of computing tasks by default. Each slow-time-scale service stage contains H=5 fast-time-scale time slots, and each episode consists of 5 slow-time-scale decision stages. All simulations were implemented in Python 3.12.3 using PyTorch 2.4.1, NumPy 2.0.1, Pandas 2.2.2, and Matplotlib 3.9.2 in a custom Python-based simulation environment. The experiments were conducted on a Windows 11 laptop equipped with an AMD Ryzen 7 6800H processor at 3.20 GHz, an NVIDIA GeForce RTX 3060 Laptop GPU with 6 GB memory, and 16 GB RAM.

The algorithm parameters of DPSO–MAPPO are set as follows. The DPSO swarm size is D=10, and the maximum number of iterations is $R_{\max}=8$. MAPPO adopts an actor–critic structure, where the actor network parameters are denoted by θ and the critic network parameters by ϕ. The discount factor is set to γ=0.95, and the PPO clipping coefficient is set to ϵ=0.2. The learning rates of the actor and critic are $5\times 10^{-4}$ and $1\times 10^{-3}$, respectively, and the batch size is 64. Unless otherwise specified in the parameter sensitivity experiments, the number of training episodes is set to 500. Other key simulation parameters are summarized in Table 2.

The baseline methods considered for comparison are as follows.

1. **Random + MAPPO:** At the slow time scale, a feasible service-point plan is generated via random search and the one with the best fitness is selected; at the fast time scale, MAPPO is adopted for task offloading decisions.
2. **DPSO + Dueling DQN:** At the slow time scale, DPSO is used to optimize the service-point migration plan, while MAPPO is replaced by Dueling DQN at the fast time scale.
3. **Random + Greedy:** At the slow time scale, random search is used; at the fast time scale, a greedy offloading policy based on instantaneous end-to-end latency is applied.

### 5.2. Convergence Performance Analysis

**Figure 3:**
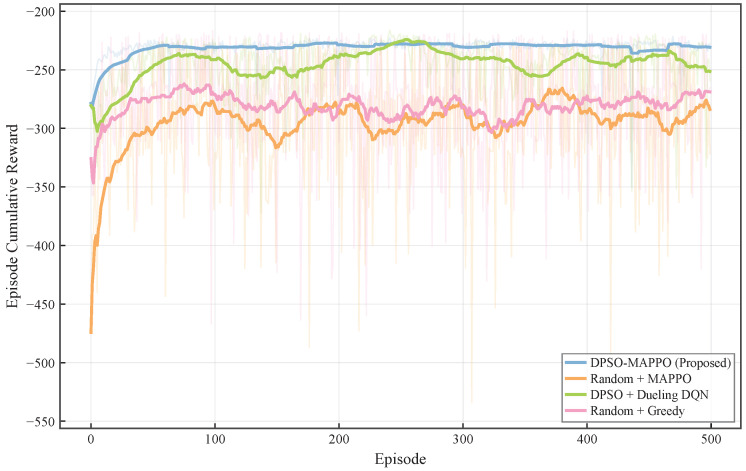
Convergence comparison of DPSO-MAPPO and benchmark algorithms.

Figure 3 depicts the training convergence curves of the proposed DPSO–MAPPO and three baseline methods. As the training episodes increase, the *episode cumulative reward* of all methods first rises rapidly and then gradually stabilizes, indicating that each method is able to learn an effective policy during training. In particular, DPSO–MAPPO achieves a faster convergence speed in the early stage and maintains the highest and most stable reward in the later stage. In contrast, Random + MAPPO relies on random search at the slow time scale, leading to an unstable quality of the service-point plan and thus a lower converged reward with larger fluctuations. Although DPSO + Dueling DQN can obtain a relatively good service-point plan via DPSO, the fast time scale adopts single-agent value-function learning, which is insufficient to fully capture the cooperative offloading interactions among multiple AGVs. Random + Greedy depends on myopic greedy decisions and lacks long-horizon policy learning capability. Therefore, the complete DPSO–MAPPO framework can simultaneously exploit the advantages of slow time-scale search and fast time-scale multi-agent learning, resulting in better convergence performance.

To further quantify the convergence behavior, Table 3 reports the convergence episode, final average reward, and reward standard deviation over the last 50 episodes. The convergence episode is defined as the first episode at which the 20-episode moving-average reward enters and remains within 5% of the final average reward. Under the dynamic non-uniform task workload setting, DPSO–MAPPO reaches the stable region after approximately 23 episodes, whereas the benchmark methods require more than 460 episodes under the same criterion. Moreover, DPSO–MAPPO achieves the highest final average reward and the smallest late-stage fluctuation, with a standard deviation of only 6.64 over the last 50 episodes. These results indicate that the proposed method achieves faster convergence and more stable late-stage performance under dynamic non-uniform task workloads.

**Figure 4:**
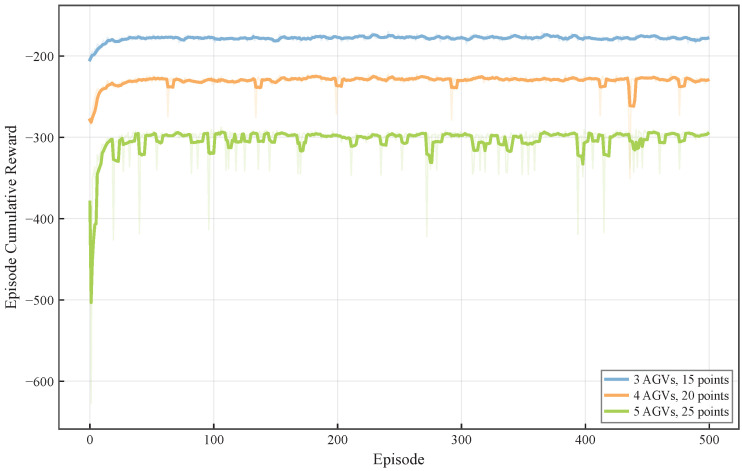
Convergence performance of DPSO-MAPPO under different system scales.

Figure 4 presents the convergence performance of DPSO-MAPPO under different system scales, where the small, medium, and large settings correspond to 3 AGVs/ 15 candidate points, 4 AGVs/20 candidate points, and 5 AGVs/25 candidate points, respectively. It can be observed that the rewards in all three cases increase rapidly at the early stage of training and gradually stabilize afterward, indicating that the proposed algorithm can achieve effective convergence under different numbers of AGVs and candidate workstations. As the system scale increases, each episode involves more AGVs, candidate workstations, and task-processing operations, leading to different levels of episode cumulative reward. Nevertheless, all curves exhibit stable convergence trends across different scales, demonstrating the adaptability and robustness of DPSO-MAPPO to variations in system scale.

**Figure 5:**
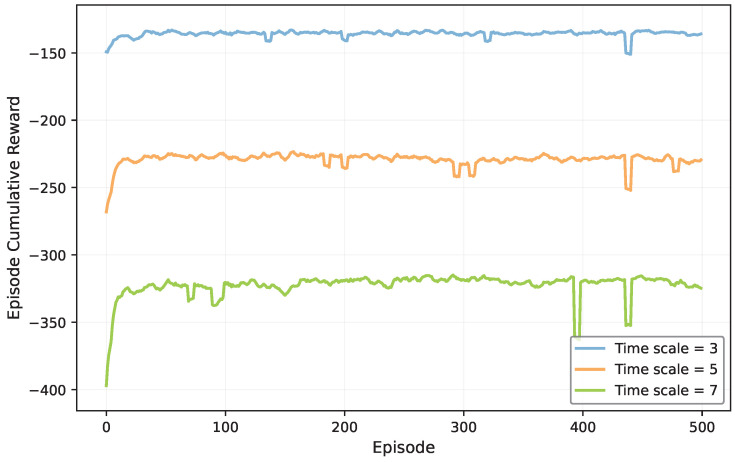
Convergence performance of DPSO-MAPPO under different time-scale settings.

Figure 5 compares the convergence performance of DPSO-MAPPO under different time-scale parameters *H*. It can be observed that the rewards in all three cases increase rapidly at the early stage of training and then gradually stabilize, indicating that the proposed algorithm achieves stable convergence under different time-scale settings. As *H* increases, each slow-timescale interval contains more fast-timescale task offloading decisions, leading to higher accumulated task processing cost and constraint pressure within an episode. Consequently, the episode cumulative reward decreases overall. Although different values of *H* change the decision horizon within each residence interval, DPSO-MAPPO maintains consistent convergence behavior across all settings, demonstrating the adaptability of the proposed dual-timescale framework to different time-scale configurations.

To further track the stability of DPSO–MAPPO under different decision intervals, Table 4 reports the stable episode, final average reward, and reward standard deviation over the last 50 episodes for H=3,5,7. The stable episode is defined as the first episode at which the 20-episode moving-average reward enters and remains within 5% of the final average reward. It can be observed that all three settings enter the stable region within about 20–21 episodes, and the standard deviations over the last 50 episodes remain limited. Although increasing *H* changes the accumulated reward scale and the length of the fast-timescale decision horizon, DPSO–MAPPO maintains stable late-stage behavior under different decision intervals.

**Figure 6:**
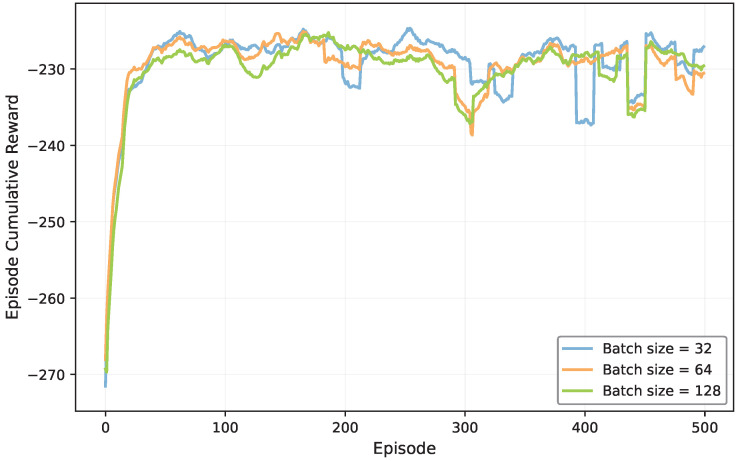
Convergence performance of DPSO-MAPPO under different batch sizes.

Figure 6 presents the convergence curves of DPSO–MAPPO under different batch sizes. All three batch-size settings can converge, but they differ in stability and convergence speed. A smaller batch size updates more frequently in the early stage and can increase the reward faster; however, it suffers from a larger variance in sample estimates and thus exhibits more noticeable fluctuations in the later stage. A larger batch size uses more samples per update and yields smoother policy changes, but its response to new samples is slower and the convergence process is relatively conservative. The default batch size achieves a better balance between convergence speed and late-stage stability, indicating that the MAPPO training configuration is well suited to the considered multi-AGV offloading scenario.

**Figure 7:**
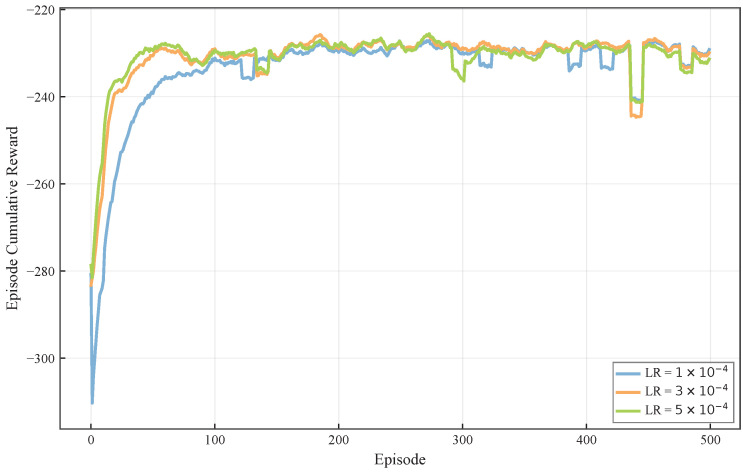
Convergence performance of DPSO-MAPPO under different learning rates.

Figure 7 shows the convergence performance under different MAPPO learning rates. Overall, all learning-rate settings lead to a rapid increase in reward at the beginning of training and then stabilize. A smaller learning rate results in smaller update steps and hence a relatively slower convergence speed in the early stage, but the curve is smoother in the later stage. A larger learning rate accelerates policy updates in the early stage but is more likely to induce local oscillations. The medium learning rate provides a good trade-off between convergence speed and stability, and is therefore chosen as the default training parameter for DPSO–MAPPO.

**Figure 8:**
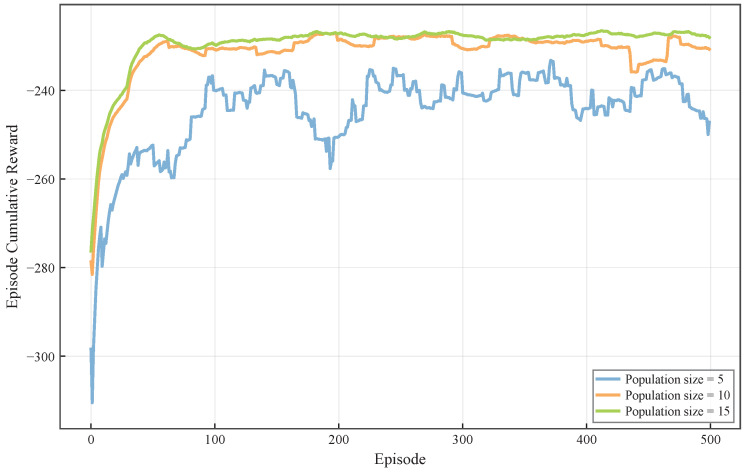
Convergence performance of DPSO-MAPPO under different DPSO population sizes.

Figure 8 presents the convergence performance of DPSO-MAPPO under different DPSO population sizes. The reward increases gradually and stabilizes in all cases, indicating that the proposed algorithm can converge effectively under different population settings. With a small population size, DPSO provides insufficient coverage of the workstation selection space, leading to a lower converged reward. As the population size increases, the search space is explored more adequately and higher-quality workstation selections can be obtained, thereby improving convergence performance. When the population size further increases, the reward improvement becomes marginal, suggesting that the performance tends to saturate. Therefore, a moderate population size can achieve a good balance between workstation search quality and convergence performance.

**Figure 9:**
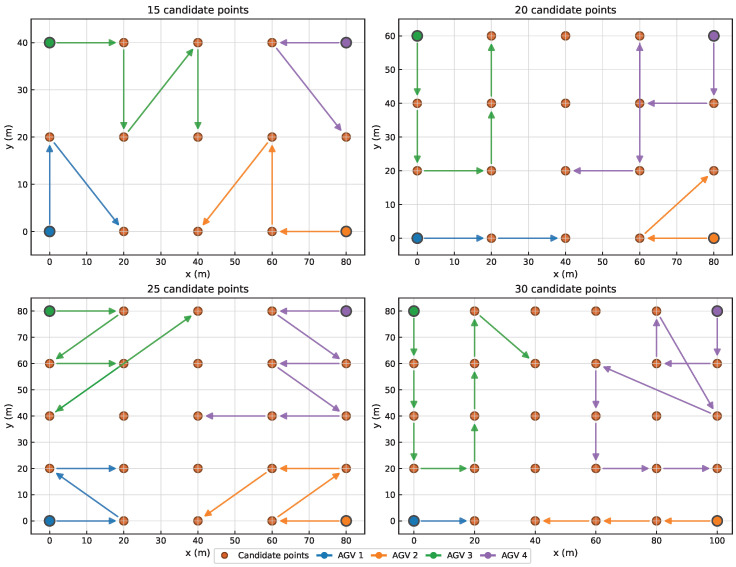
AGV service-point planning results under different numbers of candidate service points: 15, 20, 25, and 30 candidate service points. Different colors represent the trajectories of different AGVs, and the arrows indicate the migration directions.

Figure 9 presents the AGV migration trajectories generated by DPSO-MAPPO under different numbers of candidate operation points. As the number of candidate operation points increases, both the spatial distribution of operation points and the feasible decision space are gradually expanded. Nevertheless, each AGV can still form a clear cooperative trajectory and cover most candidate operation points in different regions, rather than repeatedly moving within a small local area. This indicates that, at the slow timescale, DPSO-MAPPO can effectively coordinate the operation-point selection of multiple AGVs. While satisfying the movement-distance and operation-point conflict constraints, it improves the overall service coverage of candidate operation points, thereby providing a more reasonable spatial basis for subsequent task offloading and edge collaborative computing.

### 5.3. Performance Comparison Analysis

**Figure 10:**
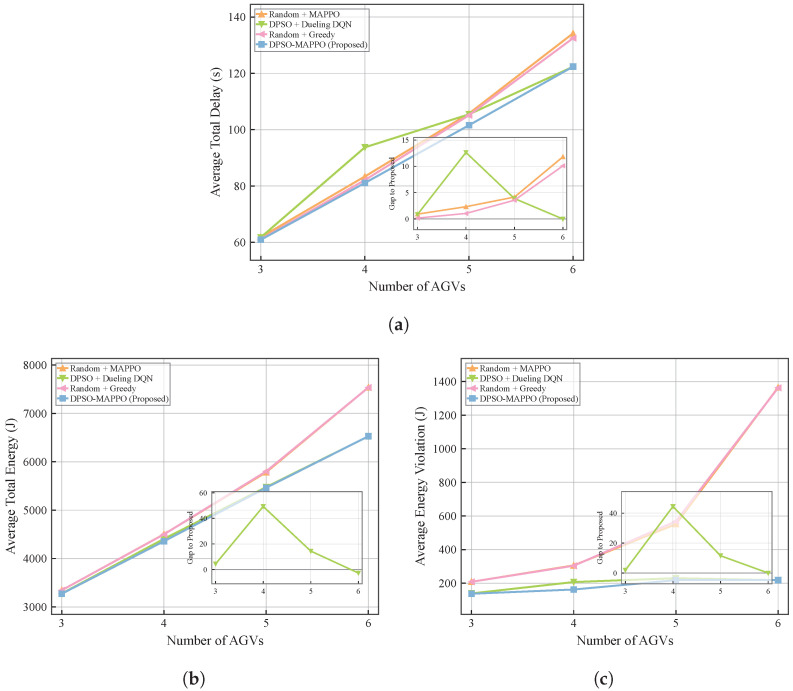
Performance comparison under different numbers of AGVs: (**a**) average total delay, (**b**) average total energy, and (**c**) average energy violation.

Figure 10 illustrates the average total delay, average total energy consumption, and average energy violation of different algorithms under varying numbers of AGVs. As the number of AGVs increases from 3 to 6, the system workload, the scale of mobility decisions, and the difficulty of multi-agent coordination rise accordingly, leading to an overall increase in both delay and energy consumption for all methods. Among them, DPSO–MAPPO (Proposed) achieves the best overall performance on all three metrics, with particularly evident advantages in energy consumption and energy violation. This is because DPSO searches for more reasonable service-point migration plans on the slow time scale, thereby reducing ineffective movements and unnecessary motion energy. Meanwhile, MAPPO performs coordinated offloading on the fast time scale according to task states, communication conditions, and remaining energy budgets, which further reduces task processing delay and suppresses energy-budget violations. In contrast, Random + MAPPO lacks effective service-point optimization; Random + Greedy relies on myopic decisions and thus lacks long-term policy learning capability; and DPSO + Dueling DQN, although capable of optimizing the slow-scale migration plan, is limited in multi-AGV coordinated offloading, making it more prone to delay/energy degradation as the system scales up.

**Figure 11:**
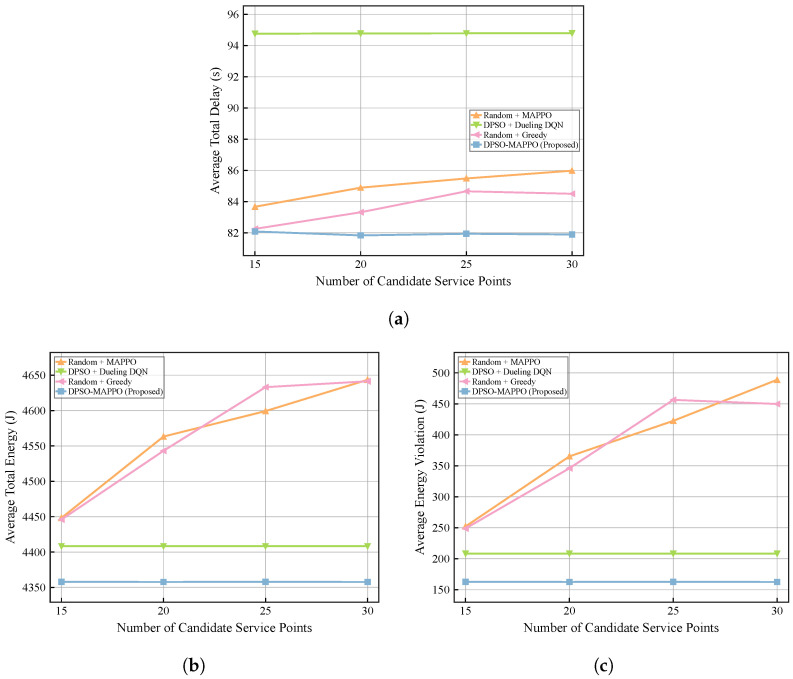
Performance comparison under different numbers of candidate service points: (**a**) average total delay, (**b**) average total energy, and (**c**) average energy violation.

Figure 11 reports the performance comparison results when the number of candidate service points varies. As the number of candidate service points increases from 15 to 30, the feasible service-point space for each AGV expands. This provides more potential high-quality service points, while meanwhile enlarging the slow-time-scale search space. As shown in Figure 11a, the average total delay of DPSO–MAPPO remains at a low level and changes only slightly with the increasing number of candidate points, indicating that the proposed algorithm can stably select effective service points from a larger candidate set. In particular, compared with DPSO + Dueling DQN, DPSO–MAPPO reduces the average total delay by 13.55%, which suggests that under the same DPSO-based service-point search mechanism, MAPPO is able to learn a more effective coordinated offloading policy among multiple AGVs. Figure 11b,c further show that DPSO–MAPPO achieves the lowest (or near-lowest) average total energy consumption and energy violation, implying that its service-point selection is not solely driven by delay minimization but also accounts for motion energy, uplink transmission energy, and local computing energy. By contrast, Random + MAPPO and Random + Greedy utilize the candidate space unstably, and increasing the number of candidates does not necessarily lead to better migration plans. DPSO + Dueling DQN performs relatively well in service-point search, yet its fast-time-scale offloading decisions are less stable than MAPPO, resulting in a weaker tradeoff between delay and energy.

**Figure 12:**
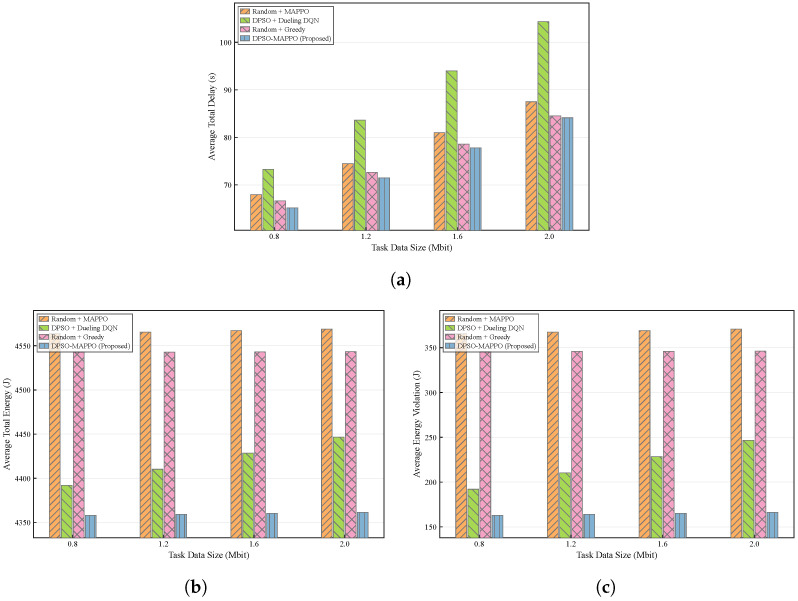
Performance comparison under different task data sizes: (**a**) average total delay, (**b**) average total energy, and (**c**) average energy violation.

Figure 12 further compares the performance under different task data sizes. As the task data size increases from 0.8 Mbit to 2.0 Mbit, the uplink transmission delay and transmission energy consumption increase, and thus the average total delay, average total energy consumption, and average energy violation of all algorithms exhibit an upward trend. From the algorithmic comparison, DPSO–MAPPO consistently maintains relatively low delay across different data sizes and shows the most pronounced advantages in total energy consumption and energy violation. This indicates that the proposed method can dynamically adjust the offloading strategy according to workload variations: when the data size is small, it exploits edge computing to reduce delay; when the data size becomes large, it avoids excessive uploading or unreasonable migrations that may cause energy surges. Random + Greedy may achieve lower instantaneous delay in some cases but lacks effective control over the long-term energy budget; Random + MAPPO is strongly influenced by random service-point selection; and DPSO + Dueling DQN is less adaptive to complex state variations under higher load. Therefore, DPSO–MAPPO demonstrates stronger delay optimization capability and better energy-constraint control under different task loads.

**Figure 13:**
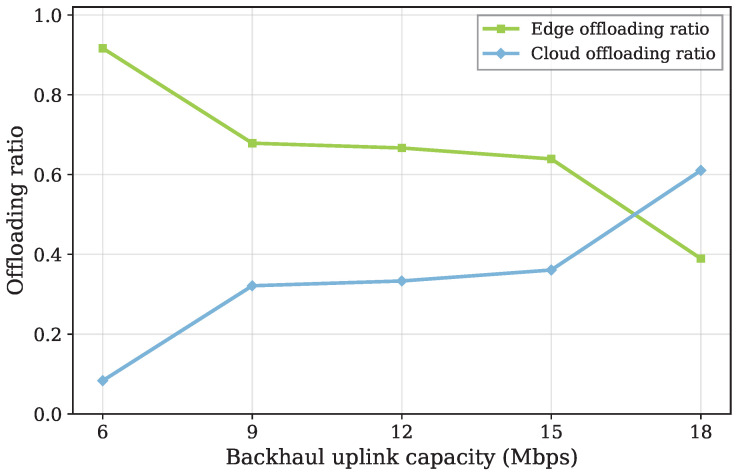
Impact of backhaul uplink capacity on edge and cloud offloading ratios.

Figure 13 shows the impact of backhaul uplink capacity on the edge and cloud offloading ratios. When the backhaul capacity is relatively low, cloud execution introduces a higher backhaul transmission cost, and most tasks are offloaded to MEC servers. As the backhaul capacity increases from 6 Mbps to 18 Mbps, the cloud offloading ratio generally increases from 0.0833 to 0.6104, while the edge offloading ratio decreases from 0.9167 to 0.3896. This result indicates that DPSO–MAPPO can adapt the edge/cloud offloading decision according to the backhaul transmission condition.

### 5.4. Ablation Study

To further evaluate the contribution of different components, Table 5 presents an ablation study of DPSO–MAPPO. Removing the DPSO module increases the total delay, total energy consumption, and energy violation, indicating that slow-timescale service-point optimization contributes to more efficient task offloading. Replacing MAPPO with Dueling DQN leads to a clear increase in total delay, which confirms the importance of multi-agent cooperative policy learning for fast-timescale offloading decisions. In addition, the Fixed-SP + MAPPO variant adopts a predefined service-point sequence instead of DPSO-based dynamic optimization and yields the largest delay, energy consumption, and energy violation, further demonstrating the necessity of adaptive service-point migration. The reward-related variants show only minor performance changes, suggesting that the learned policy remains relatively stable under moderate reward-weight perturbations in the current setting.

## 6. Conclusions

This paper studies a dual-time-scale joint optimization of AGV service-point migration and task offloading in a multi-AGV intelligent warehousing scenario under a cloud–edge–device collaborative computing architecture. A system model integrating AGV migration, wireless transmission, and local/edge/cloud computing is established, and the objective is to minimize the long-term accumulated system delay subject to task latency, AGV energy-budget, and service-point conflict constraints. To solve the resulting mixed discrete decision-making problem, a DPSO–MAPPO joint algorithm is proposed, where DPSO searches service-point migration plans on the slow time scale and MAPPO learns coordinated multi-AGV offloading on the fast time scale. Simulation results show that the proposed method generates effective migration trajectories and offloading strategies, reducing the system delay by 13.55% in a typical setting while improving total energy consumption and energy-violation control.

The results indicate that the proposed dual-time-scale design can effectively coordinate service-point migration and task offloading, thereby improving both delay performance and energy-constraint control. However, several issues remain for future work. First, more flexible time-indexed service-point sharing and dynamic collision avoidance can be considered to better support high-demand warehouse areas. Second, the communication and computation models can be extended by incorporating multi-user interference, dynamic bandwidth allocation, MEC queue evolution, and fine-grained CPU scheduling. Finally, larger-scale simulations and digital-twin or real-world AGV testbeds can be further used to validate the engineering applicability of the proposed framework.

## Figures and Tables

**Table 1:** CPU-only online inference latency of the MAPPO policy.

Metric	Mean (ms)	Std. (ms)	P95 (ms)	Min (ms)	Max (ms)
Single-agent actor inference	0.0515	0.0085	0.0621	0.0477	0.2049
Joint action generation	0.2392	0.0228	0.2700	0.2244	0.5336
End-to-end online decision	1.3793	0.2027	1.7647	1.2104	4.1483

**Table 2:** Main simulation parameter settings.

Parameter	Symbol	Value
Wireless bandwidth	*B*	4MHz
Backhaul (round-trip) rate	$R_{i}^{\mathrm{bh}}\left(t\right)$	12Mbps
Noise power	$n_{0}$	$1.0\times 10^{-13}\;W$
Task data size	$D_{i,k}\left(t\right)$	0.5–3.2Mbits
Task computing intensity	$C_{i,k}\left(t\right)$	250–1400cycles/bit
Maximum tolerable delay	$T_{k}^{\max}$	1.2–2.0s
Local CPU frequency of AGV	$f_{i}$	1.40–1.50GHz
MEC CPU frequency	$f_{m_{i}\left(t\right)}$	3.80–4.30GHz
Cloud CPU frequency	$f_{C}$	5.20GHz
AGV transmit power	$P_{i}^{\mathrm{tx}}$	0.80–0.90W
AGV motion power	$P_{i}^{\mathrm{mv}}$	80–86W
AGV moving speed	$v_{i}$	1.45–1.60m/s
Maximum moving distance	$d_{i}^{\max}$	50m
AGV energy budget	$E_{i}^{\max}$	5100–5400J

**Table 3:** Quantitative convergence comparison under dynamic non-uniform task workloads.

Algorithm	Conv. Episode	Final Avg. Reward	Std. (Last 50 Eps.)
DPSO–MAPPO (Proposed)	23	−229.73	6.64
Random + MAPPO	490	−291.06	47.93
DPSO + Dueling DQN	468	−245.62	25.32
Random + Greedy	482	−272.33	43.03

**Table 4:** Stability quantification under different decision intervals *H*.

Decision Interval *H*	Stable Episode	Final Avg. Reward	Std. (Last 50 Eps.)
3	20	−135.15	1.46
5	20	−230.24	6.82
7	21	−320.70	3.37

**Table 5:** Ablation study of different algorithmic components and reward configurations.

Variant	Avg. Total Delay (s)	Avg. Total Energy (J)	Avg. Energy Violation (J)
DPSO–MAPPO (Proposed)	81.676	4357.629	162.452
w/o DPSO	83.951	4499.839	307.748
w/o MAPPO	94.499	4407.773	207.773
Fixed-SP + MAPPO	107.588	6485.251	3115.695
w/o energy penalty	81.440	4356.407	161.229
Latency-oriented reward	81.231	4356.405	161.229
Energy-aware reward	81.841	4357.499	162.318

## Data Availability

The data presented in this study are available on request from the corresponding authors. The data are not publicly available because the research data are confidential.
